# Baseline local hemodynamics as predictor of lumen remodeling at 1-year follow-up in stented superficial femoral arteries

**DOI:** 10.1038/s41598-020-80681-8

**Published:** 2021-01-15

**Authors:** Monika Colombo, Yong He, Anna Corti, Diego Gallo, Stefano Casarin, Jared M. Rozowsky, Francesco Migliavacca, Scott Berceli, Claudio Chiastra

**Affiliations:** 1 Laboratory of Biological Structure Mechanics (LaBS), Department of Chemistry, Materials and Chemical Engineering “Giulio Natta”, Politecnico di Milano, Milan, Italy; 2 Department of Surgery, University of Florida, Gainesville, FL USA; 3 PoliToBIOMed Lab, Department of Mechanical and Aerospace Engineering, Politecnico di Torino, Turin, Italy; 4 Department of Surgery, Houston Methodist Hospital, Houston, TX USA; 5 Center for Computational Surgery, Houston Methodist Research Institute, Houston, TX USA; 6 Houston Methodist Academic Institute, Houston, TX USA; 7 Malcom Randall VAMC, Gainesville, FL USA

**Keywords:** Biomedical engineering, Risk factors, Peripheral vascular disease, Restenosis

## Abstract

In-stent restenosis (ISR) is the major drawback of superficial femoral artery (SFA) stenting. Abnormal hemodynamics after stent implantation seems to promote the development of ISR. Accordingly, this study aims to investigate the impact of local hemodynamics on lumen remodeling in human stented SFA lesions. Ten SFA models were reconstructed at 1-week and 1-year follow-up from computed tomography images. Patient-specific computational fluid dynamics simulations were performed to relate the local hemodynamics at 1-week, expressed in terms of time-averaged wall shear stress (TAWSS), oscillatory shear index and relative residence time, with the lumen remodeling at 1-year, quantified as the change of lumen area between 1-week and 1-year. The TAWSS was negatively associated with the lumen area change (ρ = − 0.75, p = 0.013). The surface area exposed to low TAWSS was positively correlated with the lumen area change (ρ = 0.69, p = 0.026). No significant correlations were present between the other hemodynamic descriptors and lumen area change. The low TAWSS was the best predictive marker of lumen remodeling (positive predictive value of 44.8%). Moreover, stent length and overlapping were predictor of ISR at follow-up. Despite the limited number of analyzed lesions, the overall findings suggest an association between abnormal patterns of WSS after stenting and lumen remodeling.

## Introduction

Lower limb peripheral artery disease (PAD) is an inflammatory disease, mainly caused by atherosclerosis, which progressively narrows the arterial lumen limiting blood supply to the leg^1^. The prevalence of this pathology, which is relatively uncommon among younger people, is continuously increasing due to population ageing^2,3^. The last updates report that a substantial proportion of the elderly population, more than 20% in 80-year-old and older individuals, suffers from PAD^2^. Notwithstanding its prevalence, its associations with mortality and morbidity, and the reduced quality of life that this disease imposes, overall PAD remains underdiagnosed and undertreated, with a spectrum of symptoms from none to severe^4–6^.

Nearly 50% of the lesions are located in the superficial femoral artery (SFA)^7^. When diagnosed, the diseased SFAs may be treated via endovascular or surgical approaches. For most of the lesions, due to its mini-invasiveness and lower risk of complications, endovascular therapy is indicated as the primary method for revascularization^8,9^. The implantation of self-expandable stents in SFAs has been a common procedure for revascularization since it improved their patency rate. However, over the last years, the optimal endovascular strategy for patients suffering from PAD in the SFA has been questioned and, specifically, the role of bare metal self-expandable stents has become a matter of great controversy^10^. Indeed, the stent implantation provides a predictable, immediate lumen gain, unachievable by many other techniques including percutaneous transluminal angioplasty and laser atherectomy^11^. However, major concerns about the durability of this outcome have been raised, specifically regarding the mid- and long-term in-stent restenosis (ISR) and stent fracture^12–14^. Indeed, at the same time, it is estimated that about 30% to 40% of stented patients will require repeated revascularization (usually surgical) at the same lesion site^13^, following vessel re-occlusion within 12 months^15^, with an ISR peak occurring between 9 and 15 months after the endovascular procedure^16^. Furthermore, it was reported that, after 2 years, the occurrence of ISR in SFA is reduced and the lesions seem more related to the evolution of atheromatous disease rather than neointimal hyperplasia^17,18^.

Besides common risk factors related to a patient’s pathological characteristics, such as disease history, smoking, and diabetes mellitus, some lesion-specific factors, including a longer lesion length and smaller vessel diameter, are potential risks of ISR^19,20^. Furthermore, as shown in previous studies on different vascular regions, in particular coronary arteries, the implantation of a stent within an artery alters the local hemodynamics, resulting in stagnation, recirculating flow, and areas subjected to low wall shear stress (WSS)^21^, which may promote the ISR development^22,23^. Recently, computational fluid dynamics (CFD) tools have been used to investigate the relationship between altered hemodynamics and restenosis in femoropopliteal artery atherosclerotic lesions treated with angioplasty or stenting^24^. A predictive statistical model of the treatment outcome at 6-month, which includes information about the artery hemodynamics immediately after intervention, was successfully proposed. However, a direct, local comparison between the luminal distribution of WSS-based descriptors and the lumen remodeling was not performed.

Accordingly, the present work aims to expand the previous analysis by investigating the impact of local hemodynamics on the vessel lumen remodeling, focusing on human SFAs treated with self-expandable stents. Specifically, patient-specific CFD simulations were performed in stented SFA models reconstructed from computed tomography (CT) images acquired at the first follow-up after stent implantation (i.e. 1-week follow-up). The local hemodynamics computed at 1-week follow-up was then related to the lumen remodeling occurring at 1-year follow-up. The analysis was conducted both at the global and local levels, taking into consideration demographic and clinical information, such as age, length of stented region, stent overlapping, and failure at 2-year follow-up, in addition to the hemodynamic descriptors and the lumen remodeling at 1-year follow-up.

## Methods

**Figure 1:**
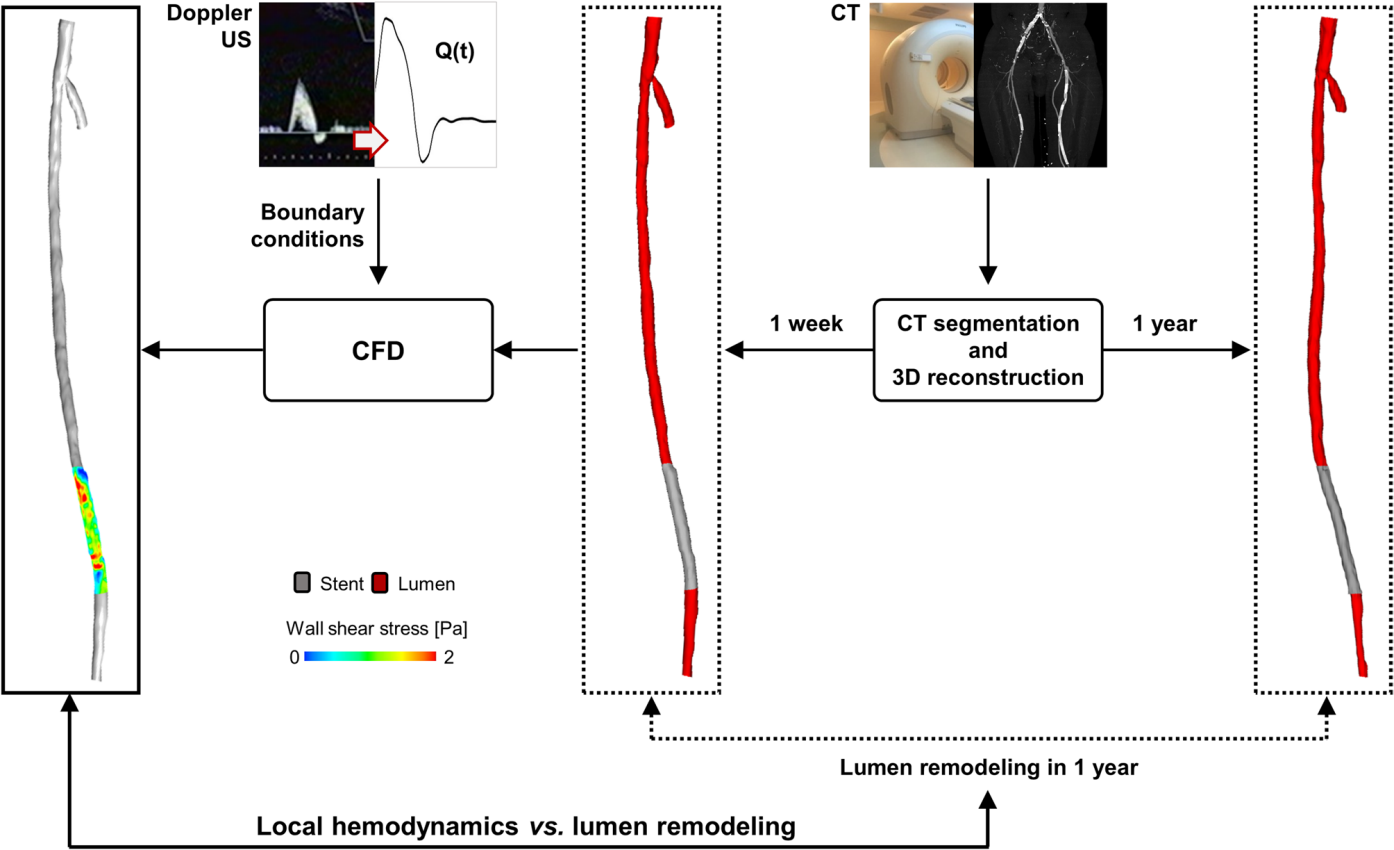
Workflow for the comparison between the local hemodynamic results at 1-week follow-up and the lumen remodeling occurring at 1-year follow-up. Starting from the computed tomography (CT) images, the vessel luminal geometries at 1-week and 1-year follow-up are reconstructed through a semi-automatic algorithm^25^. The 1-week model is used to perform computational fluid dynamics (CFD) analysis, prescribing as inlet boundary condition the velocity waveform derived from the patient’s Doppler ultrasound (US) image. Finally, the local hemodynamics is compared to lumen remodeling.

Figure 1 shows the general workflow applied to analyze the relationship between local hemodynamics and lumen remodeling in the stented region of patient-specific SFA models. The following sections detail the available clinical dataset, the methods adopted to reconstruct the SFA models, perform the CFD simulations and analyze the results.

### Clinical dataset

Consecutive patients undergoing self-expanding stent implantation and consenting to the 1-year post-operative CT scan protocol were recruited between 2007 and 2012 at the Malcom Randall VA Medical Center (Gainesville, FL, USA). Within the obtained clinical dataset, 7 patients, for a total of 10 stented lesions, presented all the imaging data necessary to perform the present study. In particular, each patient presented both CT and Doppler ultrasound (DUS) images at 1-week (baseline) and 1-year post-operative follow-up. Furthermore, the dichotomous outcome of the intervention (i.e. success/failure) was also available at 2-year follow-up, even though not supported by imaging data. The failure was defined when a new procedure of revascularization was required.

Baseline patient demographics, as well as medical history, are summarized in Table 1. The patients were all male. Their age was 62.3 ± 6.6 years (mean ± standard deviation). All lesions (A to K, Table 1) were treated with the EverFlex self-expanding stent (EV3, Medtronic, Dublin, Ireland). The stent length was 113.8 ± 38.6 mm, with five cases presenting overlapping devices. The stent overlapping was observed and defined by visual inspection of CT images and when two devices were overlapped, they were counted as a unique stent (stented region length 165.0 ± 99.6 mm). According to the 2-year follow-up, 5 out of 10 lesions failed.

**Table 1:** List of demographic and clinical characteristics.

Patient	Lesion	Age [years]	Stent length [mm]	Stented region length [mm]	Stent overlapping	Failure at 2 years
1	A	56	60	60	No	No
2	B	60	120	120	No	No
3	C	74	150 120	260	Yes	Yes
4	D	61	100 120	200	Yes	Yes
5	E	73	120 120 120	305	Yes	Yes
6	F	64	30	30	No	No
	G	64	150	150	No	No
7	H	57	40	40	No	No
	J	57	150 150	255	Yes	Yes
	K	57	120 150	230	Yes	Yes

### Human subjects/informed consent statement

This study was approved by the Institutional Review Board at the University of Florida and conformed to the Helsinki Declaration on human research of 1975, as revised in 2000. Written informed consent was obtained from the patients. No animal studies were carried out by the authors for this article.

### Three-dimensional vessel reconstruction and computational analyses

**Figure 2:**
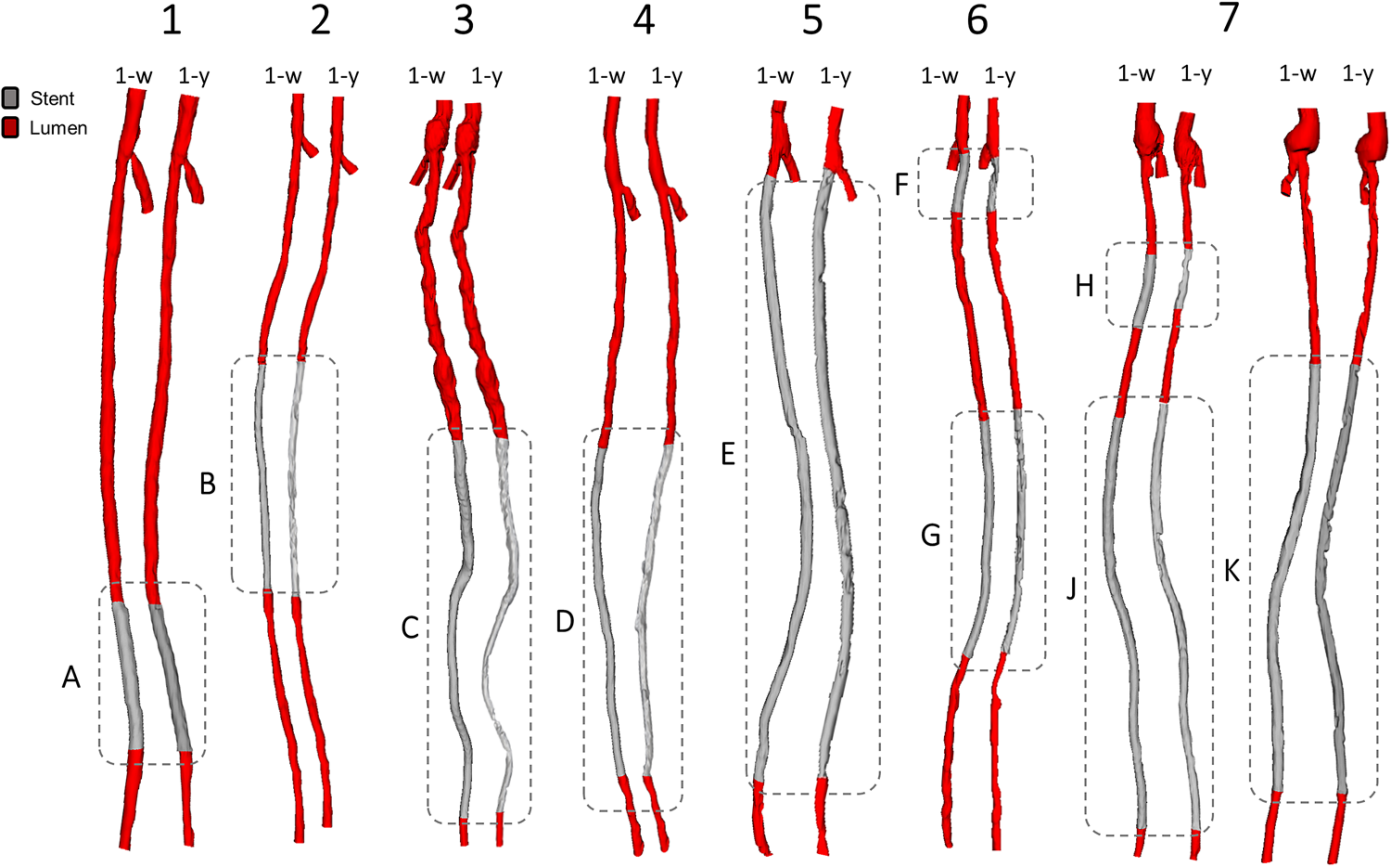
Three-dimensional reconstruction of the stented superficial femoral arteries derived from patients’ computed tomography images. For each patient, the vessel models at 1-week (1W) (i.e. baseline) and 1-year (1Y) follow-up are shown. The common femoral artery bifurcation is included in all vessel reconstructions. The lesion name is indicated besides each stented region.

Three-dimensional (3D) SFA geometrical models were reconstructed both at baseline and 1-year follow-up (Fig. 2) using a previously validated semi-automatic methodology^25^. Briefly, the CT images were segmented by means of an active contour method, based on a level set algorithm. Both the stented and the non-stented regions were segmented through calibrated thresholds. Calcifications, metallic artifacts, and thrombi within the stent were automatically removed. The common femoral bifurcation was included in all vessel reconstructions to take into account its impact on the local hemodynamics^25^.

The SFA geometrical models were discretized using tetrahedral elements with curvature-based refinement and 5 boundary layers of prismatic elements near the wall. The element size was defined according to a previously conducted mesh independence study^25^, resulting in computational grids ranging from 2,202,629 to 8,535,680 elements (SFA models with lesion B and K, respectively). The software ICEM CFD (v. 18.2, Ansys Inc., Canonsburg, PA, USA) was employed for the discretization process. Transient CFD simulations were carried out on the baseline SFA models using Fluent (v. 18.2, Ansys Inc., Canonsburg, PA, USA).

Regarding the boundary conditions, a velocity waveform derived from a patient’s DUS image was imposed at the inlet^25^. A flow-split of 67% and 33% was applied to the outlet of the SFA and the profunda femoral artery, respectively^25^. The no-slip condition was prescribed at the wall boundaries, assuming the vessel walls as rigid. Blood was modelled as a non-Newtonian fluid using the Carreau model, with a density of 1060 kg/m^3^. Details about the simulation settings are presented elsewhere^25^.

### Analysis of the results

#### Comparison between local hemodynamics and lumen area change

The local hemodynamics of the baseline SFA models was analyzed by computing three WSS-based descriptors quantifying low and/or oscillatory WSS, previously indicated as promoting factor of ISR^26^. In detail, the time-averaged WSS (TAWSS), the oscillatory shear index (OSI) and the relative residence time (RRT), were computed as follows:

1 $$TAWSS= \frac{1}{T}{\int }_{0}^{T}\left|\mathbf{W}\mathbf{S}\mathbf{S}\right|dt$$

2 $$OSI=0.5\left(1-\frac{\left|{\int }_{0}^{T}\mathbf{W}\mathbf{S}\mathbf{S} dt\right|}{{\int }_{0}^{T}\left|\mathbf{W}\mathbf{S}\mathbf{S}\right|dt}\right)$$

3 $$RRT=\frac{1}{TAWSS \cdot (1-2 \cdot OSI)}$$

where **WSS** is the WSS vector and *T* is the duration of the cardiac cycle.

As previously conducted^27^, the WSS-based data of all lesions were combined to define objective thresholds of disturbed shear stress: the 33th percentile was identified for TAWSS, the 66th percentile for OSI and RRT. The following thresholds were found: 1.05 Pa, 0.20 and 1.68 Pa^−1^ for TAWSS, OSI and RRT, respectively. Then, the percentage of surface area exposed to TAWSS (OSI and RRT) lower (higher) than the thresholds was computed and identified as TAWSS33 (OSI66 and RRT66).

**Figure 3:**
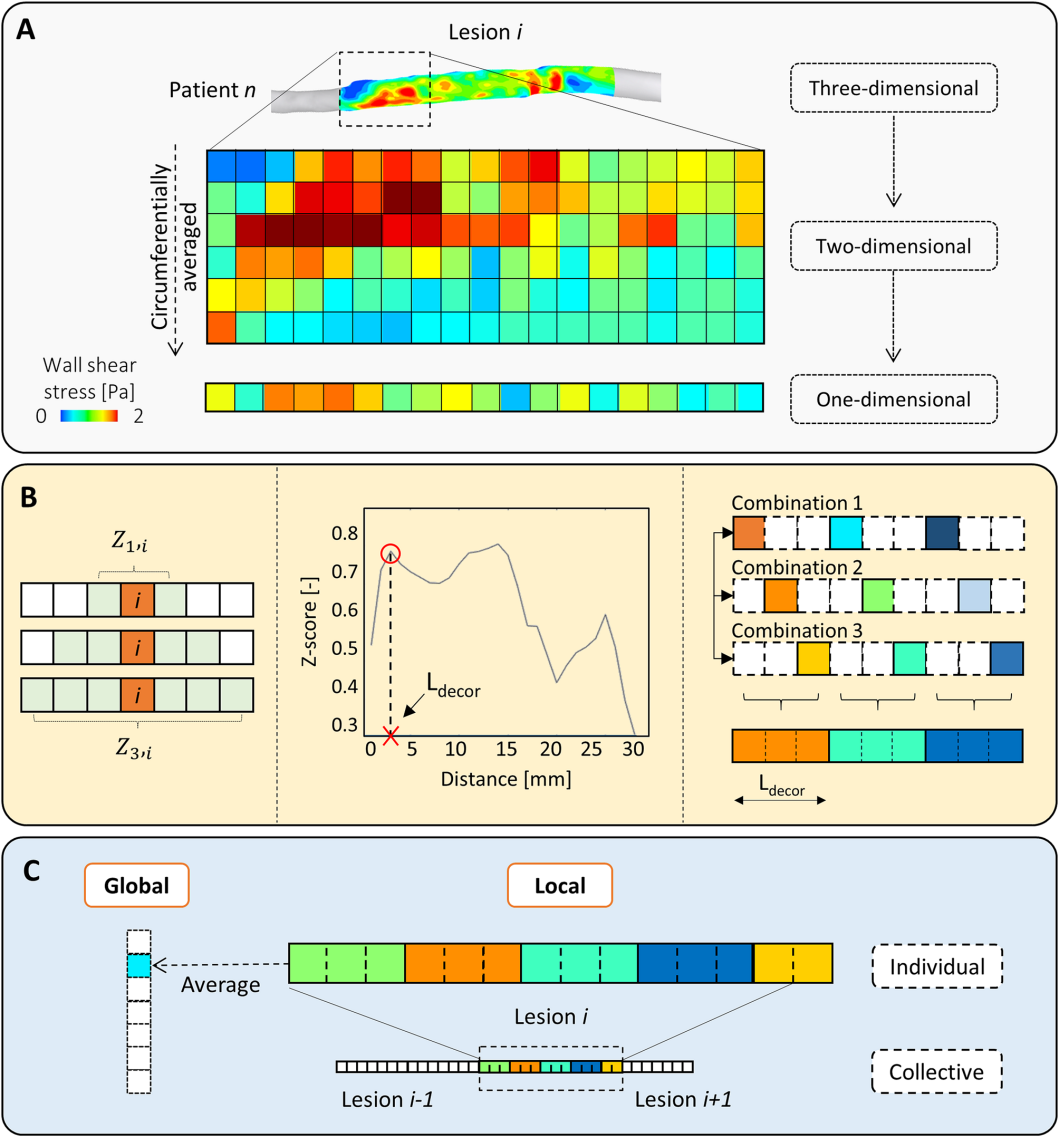
(**A**) Output of the computational fluid dynamics analysis: from the three-dimensional distribution of the wall shear stress (WSS) based variable to the one-dimensional map presenting values averaged along the circumferential direction. (**B**) Explanation of the z-score analysis: representation of the involved cells, choice of the decorrelation length and creation of the combinations with the output one-dimensional map used for the statistical analyses. In this example, the decorrelation length is 3. (**C**) Levels of investigation: (i) ‘global’, in which an averaged value is extracted from the decorrelated map of each lesion and collected with the others; (ii) ‘local’, divided into ‘individual’, in which the single lesion is considered, and ‘collective’, in which all the single lesions are collected into a unique decorrelated map.

The 3D distributions of the WSS-based descriptors along the stented portion (i.e. vessel region of interest) were also re-organized into two-dimensional (2D) maps by means of the open-source software VMTK (Orobix, Bergamo, Italy) (Fig. 3A). The 2D maps were discretized into cells of 1 mm in the axial direction and 1° in the circumferential direction. The 2D maps were then circumferentially averaged (Fig. 3A) to obtain one-dimensional (1D) maps with axial resolution of 1 mm, being conservative with respect to the CT axial resolution (lower than 1 mm in each acquisition, range = [0.87 mm, 0.98 mm]). The circumferentially averaged WSS-based data allowed the match between the local hemodynamic results at baseline and the data of lumen remodeling at 1-year post-intervention.

The lumen remodeling was quantified by calculating the lumen area change, intended as the difference between the cross-sectional lumen area at baseline and the one at 1-year follow-up. A positive (or negative) lumen area change value indicates that the remodeling at 1-year takes place in the direction of lumen area reduction (or increase). To do that, from the stented region of the 3D reconstructed vessels subdivided into 0.2 mm axially-spaced cross-sections were extracted using an automatic algorithm developed in Grasshopper (v. 5, Rhinoceros, Robert McNeel & Associates, Seattle, WA, USA). The lumen area was averaged every 1 mm in the axial direction (i.e. considering 5 cross-sections per millimeter) to obtain 1D maps with the same axial resolution as that of the WSS-based descriptors.

#### Independence of the hemodynamic results

The statistical analysis of the local association between the WSS-based descriptors and lumen remodeling requires that the data points are spatially decorrelated^28,29^. Thus, a spatial autocorrelation study was first performed to ensure the independence of the hemodynamic data points. This analysis provided a decorrelation length (L_decorr_, Fig. 3B), namely the minimum distance between the hemodynamic data points so that they can be considered as independent^28,29^. The analysis was based on the definition of an index, called z-score, derived from that defined by Peiffer et al.^28^. In particular, referring to each segment-specific result along the 1D map as a cell, for each *i*-cell the z-score was computed as:

4 $${Z}_{n,i}=\frac{{x}_{i}-{\mu }_{2n+1}}{{\sigma }_{2n+1}}$$

where *i* is the cell, ${x}_{i}$ the central value of the hemodynamic variable, $\mu$ the mean and $\sigma$ the standard deviation of the considered cells. This index was computed for each cell at different distances from the target cell, so that for each cell it is given *Z*_1,i_, *Z*_2,i_,…, *Z*_n,i_ where 1, 2, …, *n* defines the distance between the target cell and the inspected cell (Fig. 3B, left panel). For instance, for *n* = 1, ${Z}_{1,i}$, ${\mu }_{3}$ and ${\sigma }_{3}$ were evaluated only on 3 cells, for *Z*_2,i_ on 5 cells and so on. According to its definition, the z-score is greater when the value of the target cell is more distant from the mean value of the inspected cell i. Once computed for each cell, the resulting z-score was averaged based on the distance, obtaining an average Z_1_, Z_2_,…, Z_n_. The decorrelation length was then defined as the first peak in the diagram ‘averaged z-score—cell distance’ (Fig. 3B, central panel)^28,29^*.* Knowing the decorrelation length, different combinations of independent data points can be created. An average of the combinations was then calculated, thus obtaining a single 1D map (Fig. 3B, right panel), namely an array of the results for each lesion and for each variable of interest, which was used in the subsequent statistical analyses.

#### Levels of investigation and statistical analysis

The analysis of the results was divided into the following two levels (Fig. 3C): (i) ‘global’, between-stent level of analysis, in which one representative value of the hemodynamic descriptors and lumen area change was chosen for each lesion; (ii) ‘local’, within-stent level of analysis, in which the investigation was carried out at a geometrical cell level considering either each lesion separately (‘local, individual level’) or collecting all the results in only one, comprehensive case (‘local, collective level’).

Data were presented as either mean ± standard deviation or median (interquartile range), depending on the distribution. The normality of the distributions was tested by means of Kolmogorov–Smirnov test. In case of a low number of data points (e.g. global analysis with 10 data points), non-parametric tests were employed. To measure the strength and direction of the association existing between the hemodynamic descriptors and the lumen area change, the Spearman’s rank-order correlation coefficient was considered in case of monotonic distribution both at global and local, individual level. Furthermore, similarly to previous studies^30,31^, the positive predictive value (PPV) was calculated for each hemodynamic descriptor to evaluate the probability that vessel regions exposed to TAWSS33 (OSI66 and RRT66) can successfully identify corresponding regions with high lumen area change. In this analysis, the second tertile of the lumen area change distribution was used to discriminate between low/high lumen area change.

Besides the hemodynamic descriptors, the clinical information was taken into consideration. Logistic regression was performed to relate independent continuous variables to the dependent dichotomous (success-failure) variable (Tjur’s pseudo R^2^ was considered). Furthermore, a two-sided Fisher’s exact test was performed between the dichotomous variables stent overlapping and failure at 2-year follow-up.

All the statistical analyses were conducted using SPSS Statistics (v. 25, IBM, Armonk, NY, USA) and GraphPad Prism (v. 8.3.1, GraphPad Software, San Diego, CA, USA). A two-tailed, p-value < 0.05 was considered to be statistically significant.

## Results

### Spatial correlation analysis

Table 2 summarizes the lesion-specific decorrelation lengths obtained from the spatial correlation analysis (range = [2 mm, 6 mm]). For each lesion under consideration, the computed decorrelation length was different according to the hemodynamic descriptors and the most conservative value (i.e. the largest L_decorr_) was chosen for the subsequent statistical analyses.

**Table 2:** Results of the spatial correlation analysis in terms of decorrelation length (L_decorr_) and the number of decorrelated data points that are considered for the subsequent statistical analysis (N_points_).

Lesion	A	B	C	D	E	F	G	H	J	K
L_decorr_ [mm]	2	5	6	5	5	2	4	4	6	5
N_points_ [–]	30	25	43	41	62	16	37	11	43	46
TAWSS [Pa]	1.06 (0.27)	1.33 (0.42)	1.84 (0.86)	1.84 (0.72)	1.74 (1.08)	1.04 (0.31)	1.08 (0.39)	0.84 (0.25)	1.00 (0.38)	1.15 (0.30)
OSI [–]	0.09 (0.11)	0.12 (0.03)	0.33 (0.11)	0.20 (0.12)	0.11 (0.10)	0.10 (0.12)	0.09 (0.11)	0.22 (0.18)	0.23 (0.16)	0.18 (0.15)
RRT [Pa^−1^]	1.32 (0.66)	1.17 (0.41)	2.83 (2.41)	1.76 (0.75)	1.15 (0.98)	1.44 (0.73)	1.43 (0.79)	4.36 (2.93)	3.59 (3.38)	2.16 (1.24)
ΔA [mm^2^]	6.28 (1.59)	0.94 (4.19)	4.04 (5.51)	0.97 (2.23)	3.81 (10.2)	12.58 (6.16)	10.03 (5.78)	11.69 (10.20)	4.61 (2.66)	5.41 (5.38)
%TAWSS33 [–]	33%	20%	9%	5%	16%	50%	51%	100%	60%	33%
%OSI66 [–]	37%	0%	98%	41%	10%	13%	8%	36%	58%	37%
%RRT66 [–]	43%	12%	51%	20%	18%	25%	24%	64%	65%	44%

Median values (interquartile range, IQR) of time-averaged wall shear stress (TAWSS), oscillatory shear index (OSI), relative residence time (RRT), and lumen area change (ΔA), and percentage surface areas exposed to TAWSS33, OSI66 and RRT66 for each lesion under investigation.

### Global analysis

In addition to the decorrelation lengths, Table 2 reports the median values of the hemodynamic descriptors and the percentage surface areas exposed to disturbed shear computed from the decorrelated dataset of each lesion. These lesion-representative values were used in the global analysis of the results.

A statistically near-significant negative correlation was found between the median values of TAWSS and lumen area change (ρ = − 0.62, p = 0.06), suggesting that lower values of TAWSS are associated with a higher lumen area reduction. This association was statistically significant when considering the mean values of TAWSS (ρ = − 0.75, p = 0.013). Furthermore, a high positive correlation was present between the percentage surface area exposed to TAWSS33 and the lumen area change (ρ = 0.69, p = 0.026). This result indicates that larger surface areas exposed to low TAWSS are associated with a higher lumen narrowing. On the contrary, no significant associations emerged between the median values of OSI and lumen area change (ρ = − 0.40, p = 0.25) as well as between the percentage surface area exposed to OSI66 and lumen area change (ρ = − 0.15, p = 0.69). Similarly, the median values of RRT and the percentage surface area exposed to RRT66 were not significantly correlated to the lumen area change (ρ = 0.50, p = 0.143 and ρ = 0.44, p = 0.21, respectively).

**Figure 4:**
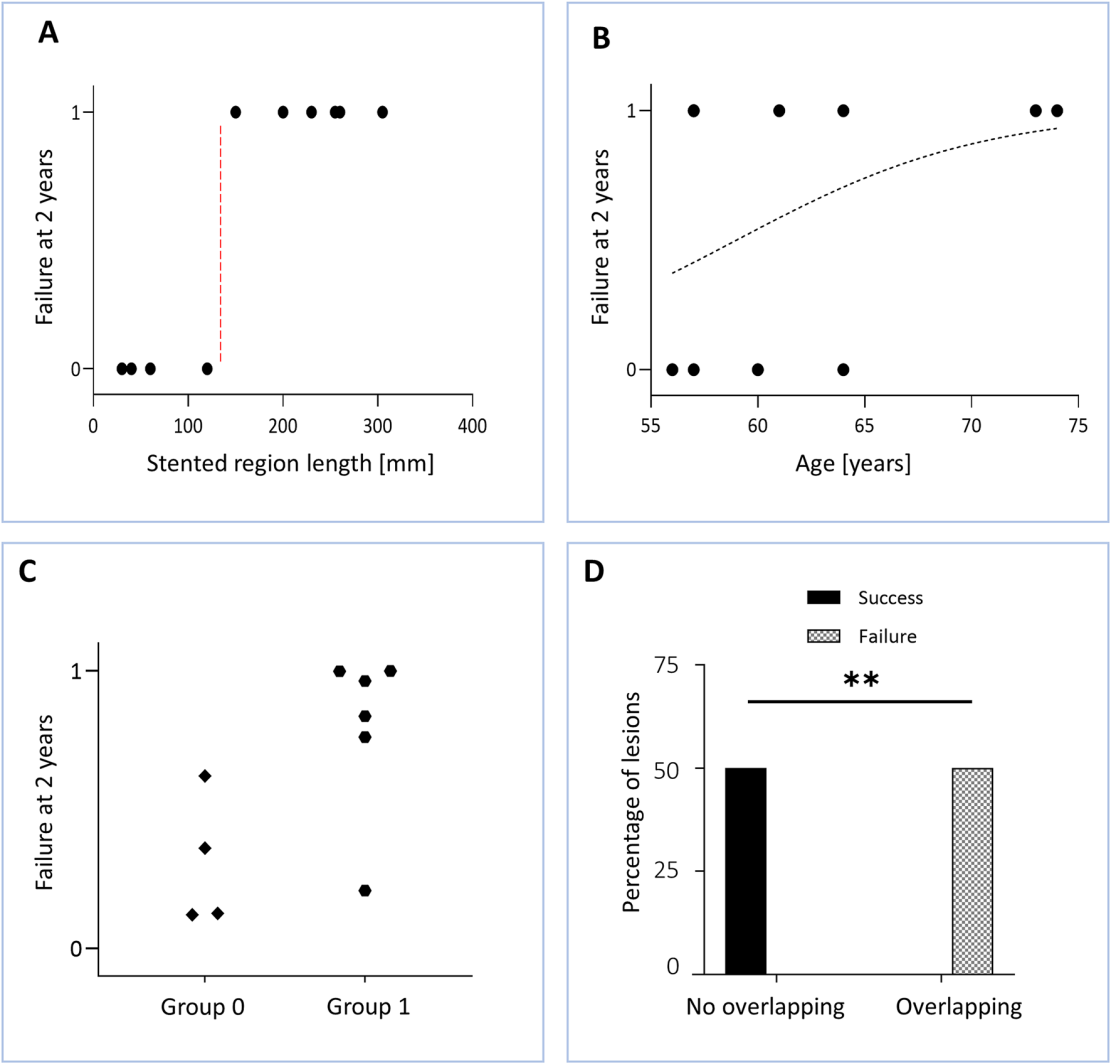
Results of the logistic regressions. (**A**) Simple logistic regression between stented region length and failure at 2-year follow-up. (**B**) Simple logistic regression between age and failure at 2-year follow-up. The dotted line represents the predicted probability of failure. (**C**) Multiple logistic regression between the hemodynamic descriptors (time-averaged wall shear stress, oscillatory shear index and relative residence time), and the predicted probability of failure at 2-year follow-up. (**D**) Result of the Fisher’s exact test that was performed to compare the dichotomous variables stent overlapping and outcome at 2-year follow-up. **p < 0.01.

Introducing the patient-specific characteristics listed in Table 1, a logistic regression (both simple and multiple) was performed to explore the relationship between the continuous variables length of the stented region (measured from the CT scans), age and hemodynamic descriptors, and the dichotomous systemic failure at 2-year post-intervention. From the simple logistic regressions, a complete separation (also called perfect prediction, i.e. the outcome variable separates completely the predictor variable) emerged between the length of the stented region with a cut-off value of 120 mm and the failure at 2-year post-intervention (Fig. 4A). The age was found not to predict the dichotomous failure (p = 0.26, R^2^ = 0.15) (Fig. 4B). Regarding the hemodynamic descriptors, the TAWSS presented a nearly statistically significant association with failure (p = 0.06, R^2^ = 0.28). The association between neither the OSI nor the RRT and the failure at 2-year post-intervention was significant (p = 0.11, R^2^ = 0.22 for OSI; p = 0.98, R^2^ < 0.0001 for RRT) (plots not shown). From the multiple logistic regression, in which all the three hemodynamic descriptors were introduced as predictors with respect to the failure at 2-year follow-up, the analysis did not show a significant difference between the success and failure groups (Fig. 4C), with a Tjur’s R^2^ equal to 0.49 (p = 0.033). Furthermore, the selected model resulted correct, according to the Hosmer–Lemeshow test (with a value of 6.83 and p = 0.55). The association between the stent overlapping and the failure was statistically significant (p = 0.008), indicating that the stent overlapping is a predictor of the treatment outcome (Fig. 4D).

### Local analysis

**Figure 5:**
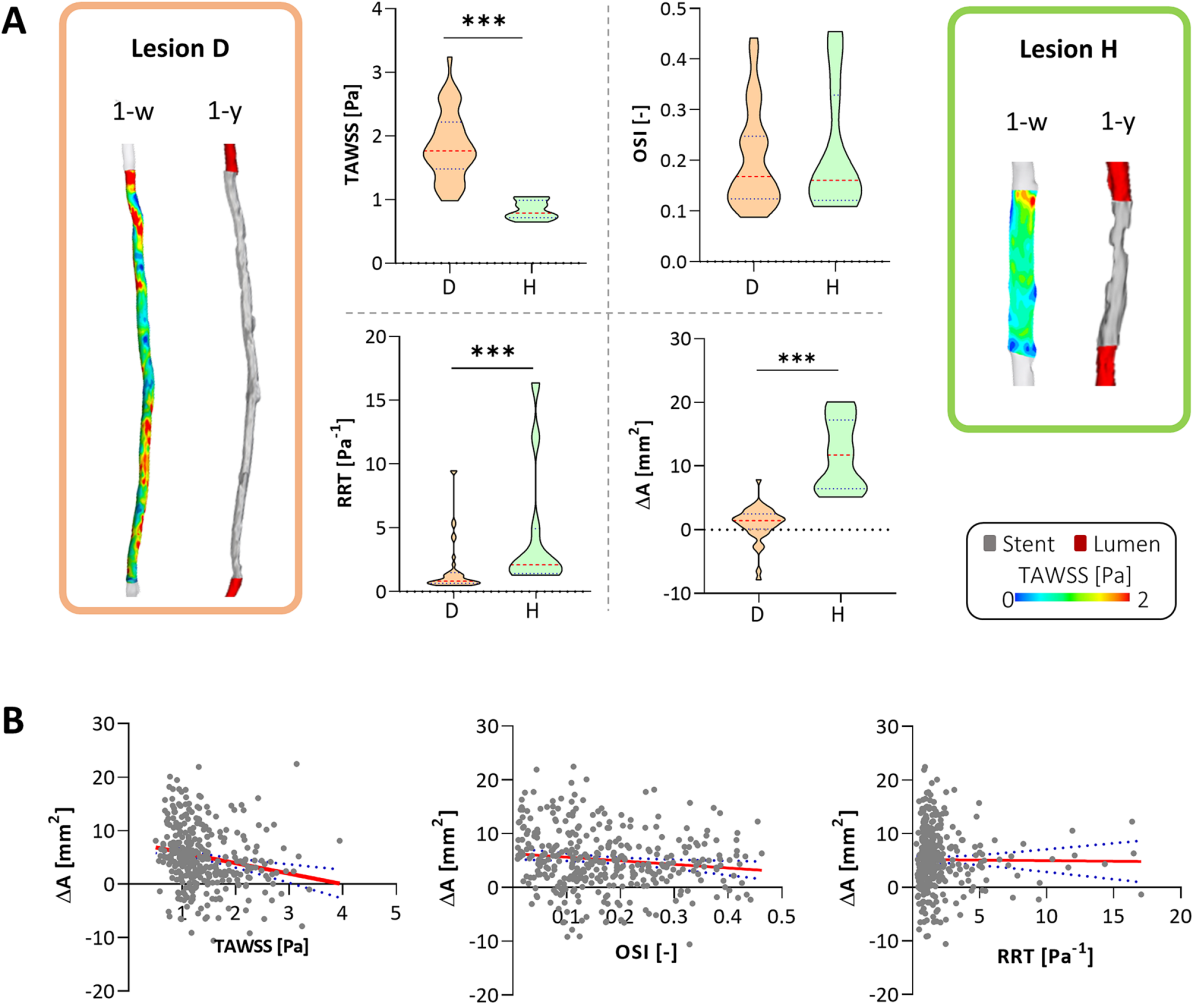
Results of the local analysis. (**A**) Comparison between the lesions presenting the lowest and the highest correlation coefficient between time-averaged wall shear stress (TAWSS) and lumen area change (lesions D and H, respectively). For both lesions, the contour maps of TAWSS computed at 1-week and the geometrical model at 1-year are shown. The violin plots, showing the distribution, median, and quartiles of each hemodynamic descriptor (TAWSS, oscillatory shear index—OSI and relative residence time—RRT) and lumen area change (ΔA), are represented. (**B**) Linear regressions between each hemodynamic descriptor and ΔA. In this individual collective analysis, all the decorrelated results were collected in a unique dataset. ***p < 0.001.

Regarding the local, individual level of analysis, no significant correlation between the hemodynamic descriptors and the lumen area change was found for most of the cases (Table 3). In two cases only, the lumen area change presented a moderate correlation with TAWSS (case A: ρ = − 0.38, p = 0.04; case H: ρ = − 0.64, p = 0.02). The two lesions presenting the lowest and the highest correlation coefficient between TAWSS and lumen area change (i.e. lesions D and H, respectively) were also taken as examples to show the contour maps of TAWSS along the stented region at baseline and the corresponding vessel geometry at 1-year follow-up as well as the distributions of the variables of interest (Fig. 5A). The two cases were characterized by statistically different distributions of TAWSS (p < 0.0001), RRT (p = 0.0005) and lumen area change (p < 0.0001), according to the Mann–Whitney rank test. Conversely, no significant differences were found for the distributions of OSI of the two lesions. Lesion H, which presented lower values of TAWSS, showed a pronounced lumen narrowing as compared to lesion D.

**Table 3:** Results of the local individual analysis, in which Spearman’s correlation coefficients between hemodynamic descriptor and lumen area change are shown for each lesion and for each hemodynamic descriptor. Bold indicate significant statistical results.

Lesion	A	B	C	D	E	F	G	H	J	K
TAWSS	− 0.38*	− 0.16	0.29	− 0.01	0.17	0.6	0.17	s− 0.64*	− 0.16	− 0.08
OSI	0.07	− 0.19	0.22	0.01	− 0.12	− 0.4	− 0.33	0.14	− 0.13	0.14
RRT	0.34	0.09	0.02	− 0.02	− 0.17	− 0.6	− 0.23	0.39	0.1	0.17

*p < 0.05.

Regarding the local, collective level of analysis, the Spearman’s correlation was performed to examine the relationship between the hemodynamic descriptors. A negligible negative correlation was found between TAWSS and OSI (ρ = − 0.21). On the contrary, as expected, significant high correlations were present between TAWSS and RRT (ρ = − 0.69, p < 0.0001) and between OSI and RRT (ρ = 0.77, p < 0.0001). Spearman’s correlation and linear regression analyses between the hemodynamic descriptors and the lumen area change revealed that the strongest association was present between TAWSS and lumen area change, as observed in the global analysis (“Global analysis” section). In particular, a negative correlation was found (ρ = − 0.28, p < 0.0001). Moreover, the regression line (Fig. 5B, left panel) had a negative slope, indicating that for higher values of TAWSS, the lumen narrowing reduces. A weak association emerged between OSI and lumen area change (ρ = − 0.15, p = 0.005), whereas no significant relationship was found between RRT and lumen area change (ρ = 0.07, p = 0.184). Finally, to evaluate the predictive power of the hemodynamic descriptors, the PPV was quantified. The PPV was 44.8%, 28.6% and 35.9% for the TAWSS, OSI and RRT, respectively.

## Discussion

ISR in SFAs is a complex multi-factorial process widely affecting the outcome of the stenting procedure^32^. Among the different factors promoting ISR, this study investigated the impact of the altered hemodynamics after stent implantation on vessel lumen remodeling. Specifically, a computational approach, based on the 3D reconstruction of patient-specific SFA geometrical models and on CFD simulations^25^, was adopted to relate the local hemodynamics at baseline, expressed in terms of WSS-based descriptors, with the lumen remodeling at 1-year follow-up. The findings of this study suggest that in human SFA lesions an association between the TAWSS at baseline and the lumen remodeling at 1-year follow-up exists. Specifically, (i) lower baseline values of TAWSS were associated with higher lumen area reduction, and (ii) larger surface areas exposed to lower baseline TAWSS were associated with higher lumen area reduction. On the contrary, no statistically significant correlations were present between the other analyzed hemodynamic descriptors (i.e. OSI and RRT) and lumen area change.

Although computational approaches have been previously applied to investigate the influence of local hemodynamics on ISR in other vascular regions, in particular in coronary arteries^23,33,34^, their application to lower-limb peripheral arteries has been limited. Until now, a few studies have focused on the influence of curvature and tortuosity on atherosclerosis^35,36^, or on the effect of wall motion^37^ and leg flexion^38,39^ on femoral artery hemodynamics. Only in a recent study by Gökgöl and colleagues^24^, CFD simulations were performed in image-based femoro-popliteal artery models to analyze the local hemodynamics under straight and flexed leg positions and relate the WSS-based descriptors to the occurrence of restenosis at 6-month after angioplasty or stenting procedure. A logistic regression model including a combination of different hemodynamic descriptors and the treatment method was built showing an accuracy of 80% in predicting the 6-month outcome. However, none of the WSS-based descriptors was able to explain the occurrence of restenosis alone. The imaging modality used for the vessel reconstruction (i.e. 2D angiography) and the lack of patient-specific boundary conditions may have affected the hemodynamic results in that study. More importantly, the analyzed arteries were only classified in two groups, namely non-restenosed and restenosed arteries, based on the 6-month outcome but a local quantification of the lumen remodeling was not conducted. On the contrary, the present study provided a local quantitative comparison between the hemodynamic descriptors and the lumen area change focusing exclusively on SFA segments treated with self-expanding stents. The use of CT images for the 3D vessel reconstruction allowed obtaining more accurate SFA geometries, being at the same time less invasive. Furthermore, patient-specific boundary conditions, derived from DUS images, were here applied to the CFD models.

Before investigating the link between local hemodynamics and lumen remodeling, a preliminary spatial correlation analysis was carried out to eliminate the data points that could potentially be auto-correlated or mutually influenced^29,40^. Indeed, several statistical approaches are commonly used under the assumption that the measured outcomes are independent of each other. Nonetheless, it is very liable that, especially in spatial data, some or all outcome measures exhibit spatial autocorrelation^29,40^. A previously described methodology^28^ was here adapted to circumferentially averaged 1D data in order to create an ensemble of decorrelated and independent data points. The spatial correlation analysis highlighted that the minimum distance at which two data points could be considered as decorrelated is lesion-specific and should be evaluated case-by-case. The main explanation for this could be that the hemodynamic descriptors are directly related to the geometrical shape of the specific arterial model.

To explore if a correlation exists between each hemodynamic descriptor and the lumen area change, the statistical analyses were conducted both at a global and local level. The global findings, obtained considering one representative value for each lesion, were partially confirmed by the local analyses. In particular, also at the local, collective level, a negative correlation was found between the TAWSS and the lumen area change. The lower correlation coefficient as compared to that found in the global analysis may be explained by the higher degree of uncertainty and the inaccuracy that is introduced when a deeper, local investigation level is reached, as observed in previous works^41,42^. At this level of analysis, the total error affecting the statistical results was given by the sum of multiple factors, including (i) the 3D vessel reconstruction error, associated to the limited CT resolution, (ii) the uncertainty related to the inlet boundary condition, due to its derivation from DUS images, (iii) the computational error and (iv) the approximations related to the post-processing of the results, in particular the circumferential averaging of the results, which resulted in 1D maps that did not consider how the hemodynamic descriptors and the lumen remodeling varied circumferentially along each segment of interest.

Despite the previous considerations, the local level of analysis enabled the establishing of the PPV for the lumen remodeling for each hemodynamic descriptor. While the PPV was low for the high OSI and RRT (28.6% and 35.9%, respectively), a PPV of 44.8% was found for the low TAWSS. This predictive value for the low TAWSS seems promising, indicating that the low TAWSS is a good predictor for local lumen narrowing. In fact, although a direct comparison of this index with other computational studies on SFAs and ISR is not possible, previous computational hemodynamics studies focusing on plaque progression in coronary arteries reported a lower^31,43^ or similar PPVs^30^.

Additionally, this study provided insights into the relationship between the hemodynamic descriptors, some demographic and clinical variables (i.e. patient’s age, length of the stented region, and stent overlapping) and the success or failure of the endovascular procedure at 2-year follow-up. No significant relationship was found between patients’ age and treatment failure at 2-year post-intervention, in accordance with a previous work^44^. Conversely, the length of the stented region and the presence of stent overlapping were predictors of the treatment failure, coherently with previous findings in other vascular segments^45^.

Some limitations might affect the findings of this study. The statistical power of the used models was limited by the dimension of the clinical dataset. In particular, at the global level only 10 points per analysis were considered, possibly affecting the statistical power of the used models and the significance of the correlations. However, a local, collective level of analysis was also taken into account. At this level, the analyses counted more than 350 independent points, and the resulting findings were aligned with those of the global analyses, thus supporting and extending that level of investigation. Moreover, similarly to a previous study^24^, the SFA models did not include the stent geometry because of the limited resolution of the clinical images used for the 3D reconstruction, which did not allow detecting the stent struts. Although the effect of stent struts, stent overlapping and malapposition on hemodynamics is not taken into account, the relationship between hemodynamics and lumen remodeling emerged here. Finally, the local hemodynamics of the stented SFAs was investigated under the assumption of rigid walls and straight leg configuration, assuming that the leg movement did not markedly affect the hemodynamic results of the SFA segment. Future patient-specific studies may include the effect of lower limb movement by adopting the methodology recently implemented in an idealized moving-boundary CFD model of femoropopliteal artery^39^.

## Conclusions

In this study, the impact of local hemodynamics on lumen remodeling was evaluated in human SFA lesions treated with self-expanding stents. The overall findings showed an association between abnormal patterns of WSS at baseline and lumen narrowing at 1-year follow-up. Specifically, lower TAWSS values and larger areas exposed to low TAWSS were associated with higher lumen area reduction. Conversely, OSI and RRT did not show any significant correlation with the lumen area change. Furthermore, the low TAWSS was a better predictive marker (i.e. higher PPV) of lumen remodeling as compared to OSI and RRT. In addition to the hemodynamic analysis, clinical variables such as the length of stented region and the presence of stent overlapping in those lesions treated with multiple stents were recognized as predictors of the treatment failure at 2-year follow-up. Despite these promising results, these associations need to be confirmed on a wider clinical dataset, with the final goal of defining stronger clinical predictors for ISR in SFAs. In this perspective, the presented workflow might serve as a clinical tool to predict the outcomes of a stenting procedure in SFA based on the hemodynamics analysis.

## References

[1] Krishna SM, Moxon JV, Golledge J (2015). A review of the pathophysiology and potential biomarkers for peripheral artery disease. Int. J. Mol. Sci..

[2] Shu J, Santulli G (2018). Update on peripheral artery disease: Epidemiology and evidence-based facts. Atherosclerosis.

[3] Nakamura M, Jaff MR, Settlage RA, Kichikawa K, RELIABLE Investigators (2018). Nitinol self-expanding stents for the treatment of obstructive superficial femoral artery disease: Three-year results of the RELIABLE Japanese multicenter study. Ann. Vasc. Dis..

[4] Fowkes FGR (2013). Comparison of global estimates of prevalence and risk factors for peripheral artery disease in 2000 and 2010: A systematic review and analysis. Lancet.

[5] Fowkes G (2008). Ankle brachial index combined with Framingham risk score to predict cardiovascular events and mortality: A meta-analysis. JAMA J. Am. Med. Assoc..

[6] Olin JW, White CJ, Armstrong EJ, Kadian-Dodov D, Hiatt WR (2016). Peripheral artery disease: Evolving role of exercise, medical therapy, and endovascular options. J. Am. Coll. Cardiol..

[7] Shaker A, Rahim A, El Kashef O, Gad A (2016). Balloon angioplasty versus stenting of sequential tandem lesions in superficial femoral and popliteal arteries. Egypt. J. Surg..

[8] Wiseman JT (2017). Endovascular versus open revascularization for peripheral arterial disease. Ann. Surg..

[9] AbuRahma AF (2018). When are endovascular and open bypass treatments preferred for femoropopliteal occlusive disease?. Ann. Vasc. Dis..

[10] Thukkani AK, Kinlay S (2015). Endovascular intervention for peripheral artery disease. Circ. Res..

[11] Gray BH, Buchan JA (2016). The treatment of superficial femoral artery in-stent restenosis: The jury is still out. JACC Cardiovasc. Interv..

[12] Babaev AA, Kotwal A, Zavlunova S, Telis A (2013). Stent fractures in the superficial femoral artert and restenosis: How strong is the association?. J. Am. Coll. Cardiol..

[13] Golzar JA (2017). Long in-stent restenosis of the superficial femoral artery successfully treated using OCT-guided directional atherectomy. Vasc. Dis. Manag..

[14] Abdoli S, Katz S, Ochoa C (2019). Long-term patency and clinical outcomes of nitinol stenting for femoropopliteal atherosclerotic disease. Ann. Vasc. Surg..

[15] Walker C (2015). Interventional treatment of superficial femoral artery in-stent restenosis. Vasc. Dis. Manag..

[16] Kim W, Choi D (2018). Treatment of femoropopliteal artery in-stent restenosis. Korean Circ. J..

[17] Laird JR (2010). Nitinol stent implantation versus balloon angioplasty for lesions in the superficial femoral artery and proximal popliteal artery: Twelve-month results from the RESILIENT randomized trial. Circ. Cardiovasc. Interv..

[18] Dake MD (2011). Paclitaxel-eluting stents show superiority to balloon angioplasty and bare metal stents in femoropopliteal disease: Twelve-month zilver PTX randomized study results. Circ. Cardiovasc. Interv..

[19] Mehran R (1999). Angiographic patterns of in-stent restenosis classification and implications for long-term outcome. Circulation.

[20] Scheinert D (2005). Prevalence and clinical impact of stent fractures after femoropopliteal stenting. J. Am. Coll. Cardiol..

[21] Chiastra, C., Dubini, G. & Migliavacca, F. Hemodynamic perturbations due to the presence of stents. In *Biomechanics of Coronary Atherosclerotic Plaque* 257–278 (Elsevier, Amsterdam, 2020) 10.1016/B978-0-12-817195-0.00011-1

[22] Wang J (2018). Endovascular stent-induced alterations in host artery mechanical environments and their roles in stent restenosis and late thrombosis. Regen. Biomater..

[23] Ng J (2017). Local hemodynamic forces after stenting: Implications on restenosis and thrombosis. Arterioscler. Thromb. Vasc. Biol..

[24] Gökgöl C, Diehm N, Räber L, Büchler P (2019). Prediction of restenosis based on hemodynamical markers in revascularized femoro-popliteal arteries during leg flexion. Biomech. Model. Mechanobiol..

[25] Colombo M (2020). Computing patient-specific hemodynamics in stented femoral artery models obtained from computed tomography using a validated 3D reconstruction method. Med. Eng. Phys..

[26] Van Der Heiden K (2013). The effects of stenting on shear stress: Relevance to endothelial injury and repair. Cardiovasc. Res..

[27] De Nisco G (2019). The atheroprotective nature of helical flow in coronary arteries. Ann. Biomed. Eng..

[28] Peiffer V, Bharath AA, Sherwin SJ, Weinberg PD (2013). A novel method for quantifying spatial correlations between patterns of atherosclerosis and hemodynamic factors. J. Biomech. Eng..

[29] Rowland EM, Mohamied Y, Yean Chooi K, Bailey EL, Weinberg PD (2015). Comparison of statistical methods for assessing spatial correlations between maps of different arterial properties. J. Biomech. Eng..

[30] Hoogendoorn A (2020). Multidirectional wall shear stress promotes advanced coronary plaque development: Comparing five shear stress metrics. Cardiovasc. Res..

[31] Stone PH (2012). Prediction of progression of coronary artery disease and clinical outcomes using vascular profiling of endothelial shear stress and arterial plaque characteristics: The PREDICTION study. Circulation.

[32] Waksman R, Iantorno M (2018). Refractory in-stent restenosis: Improving outcomes by standardizing our approach. Curr. Cardiol. Rep..

[33] Morlacchi S (2011). Hemodynamics and In-stent restenosis: Micro-CT images, histology, and computer simulations. Ann. Biomed. Eng..

[34] Mongrain R, Rodés-Cabau J (2006). Role of shear stress in atherosclerosis and restenosis after coronary stent implantation. Rev. Española Cardiol..

[35] Li X (2019). Tortuosity of the superficial femoral artery and its influence on blood flow patterns and risk of atherosclerosis. Biomech. Model. Mechanobiol..

[36] Xu P (2016). Patient-specific structural effects on hemodynamics in the ischemic lower limb artery. Sci. Rep..

[37] Kim Y-H (2008). Hemodynamic analysis of a compliant femoral artery bifurcation model using a fluid structure interaction framework. Ann. Biomed. Eng..

[38] Desyatova A (2018). Effect of aging on mechanical stresses, deformations, and hemodynamics in human femoropopliteal artery due to limb flexion. Biomech. Model. Mechanobiol..

[39] Colombo M (2020). Impact of lower limb movement on the hemodynamics of femoropopliteal arteries: A computational study. Med. Eng. Phys..

[40] Sokal RR, Oden NL (1978). Spatial autocorrelation in biology: 1. Methodology. Biol. J. Linn. Soc..

[41] Gallo D (2018). Segment-specific associations between local haemodynamic and imaging markers of early atherosclerosis at the carotid artery: An in vivo human study. J. R. Soc. Interface.

[42] Steinman DA, Pereira VM (2019). How patient specific are patient-specific computational models of cerebral aneurysms? An overview of sources of error and variability. Neurosurg. Focus.

[43] Rikhtegar F (2012). Choosing the optimal wall shear parameter for the prediction of plaque location—A patient-specific computational study in human left coronary arteries. Atherosclerosis.

[44] Cassese S (2018). Incidence and predictors of recurrent restenosis after drug-coated balloon angioplasty for restenosis of a drug-eluting stent: The ICARUS cooperation. Rev. Española Cardiol..

[45] Räber L (2010). Impact of stent overlap on angiographic and long-term clinical outcome in patients undergoing drug-eluting stent implantation. J. Am. Coll. Cardiol..

